# Age-Related Increase in Skull Vibration-Induced Nystagmus Slow-Phase Velocity in a Clinical Population with Normal vHIT and VEMP: A Retrospective Cross-Sectional Analysis

**DOI:** 10.3390/audiolres16050128

**Published:** 2026-08-31

**Authors:** Melissa Blanco-Pareja, Ruth Escudero-Medina, Nicolás Pérez-Fernández

**Affiliations:** Department of Otorhinolaryngology, Clínica Universidad de Navarra, 28027 Madrid, Spain

**Keywords:** skull vibration-induced nystagmus, vestibular asymmetry, vertigo, dizziness, vHIT, VEMP, presbyvestibulopathy, vestibular aging

## Abstract

**Background/Objectives:** Skull vibration-induced nystagmus (SVIN) is a reproducible marker of vestibular asymmetry, but its relationship with age in patients with normal conventional tests remains unclear. We evaluated whether age is associated with the presence of SVIN and with its slow-phase velocity (SVIN-SPV). **Methods:** In this retrospective cross-sectional analysis, 164 patients with dizziness or vertigo and normal video head impulse test (vHIT) and VEMPs were studied (107 SVIN-positive, 57 SVIN-negative). Logistic regression tested whether age predicted SVIN positivity; among the 107 positive patients, SVIN-SPV was analyzed by correlation and multivariable linear regression. **Results:** Age did not predict a positive SVIN (odds ratio 1.01 per year, *p* = 0.28), whereas male sex did (odds ratio 2.31, *p* = 0.02). Among positive patients, mean SVIN-SPV was 3.0°/s and correlated with age (r = 0.25, *p* = 0.009); age was the only significant covariate in multivariable regression (β = 0.035°/s per year, *p* = 0.010), and patients aged ≥60 years showed higher SVIN-SPV (*p* = 0.028). **Conclusions:** Thus, age was not associated with whether SVIN was present but with its intensity once present, compatible with possible age-related changes in vestibular asymmetry or central compensation, although the underlying mechanism was not directly assessed; these findings should not be generalized to physiological vestibular aging.

## 1. Introduction

Skull vibration-induced nystagmus (SVIN) is a simple and reproducible clinical marker of vestibular asymmetry. Its directional consistency and sensitivity to unilateral vestibular disorders support its use both at the bedside and in standardized laboratory protocols [1,2,3,4]. SVIN is thought to reflect interlabyrinthine asymmetry under dynamic stimulation rather than absolute vestibular output, which distinguishes its interpretation from that of other vestibular tests [5,6,7,8,9,10].

Age-related changes in SVIN have been reported, but the findings remain inconsistent. Aging is associated with a decline in peripheral vestibular responses and central adaptive capacity; however, SVIN may behave differently because it is governed by at least two competing mechanisms: reduced vestibular drive on the one hand, and increased side-to-side asymmetry or less efficient central suppression on the other [11,12,13]. Accordingly, reduced, preserved, and even enhanced SVIN responses have all been described in older individuals, depending on population characteristics and the specific outcome measure (e.g., slow-phase velocity versus beat frequency).

The clinical interpretation of SVIN is particularly challenging in patients with normal function on standard tests such as the video head impulse test (vHIT) and vestibular evoked myogenic potentials (VEMPs). In this setting, typically characterized by a low-amplitude SVIN, the response may represent either nonspecific variability or a subtle asymmetry that becomes more apparent with age-related changes in compensation and sensory reweighting. The extent to which age influences SVIN independently of other clinical factors—sex, diagnostic category, or spontaneous nystagmus—remains insufficiently defined.

The present study therefore aimed to assess, in a consecutive clinical population with normal vHIT and VEMP responses, both whether age is associated with the presence of a positive SVIN in the full eligible population and whether age is associated with SVIN slow-phase velocity (SVIN-SPV) among SVIN-positive patients, while assessing the potential confounding effects of sex, diagnostic category, and spontaneous nystagmus [14]. We deliberately framed the analysis as cross-sectional and hypothesis-generating rather than as a demonstration of physiological vestibular aging.

## 2. Materials and Methods

### 2.1. Study Design and Ethics

It was a secondary analysis of anonymized clinical data acquired during routine care between 1 November 2022 and 31 October 2024, without intervention. The study was conducted in accordance with the Declaration of Helsinki, and the Research Ethics Committee of Clínica Universidad de Navarra granted a waiver of individual informed consent given the retrospective design and the use of fully anonymized data (protocol code 2024.769).

### 2.2. Participants and Diagnostic Classification

**Figure 1:**
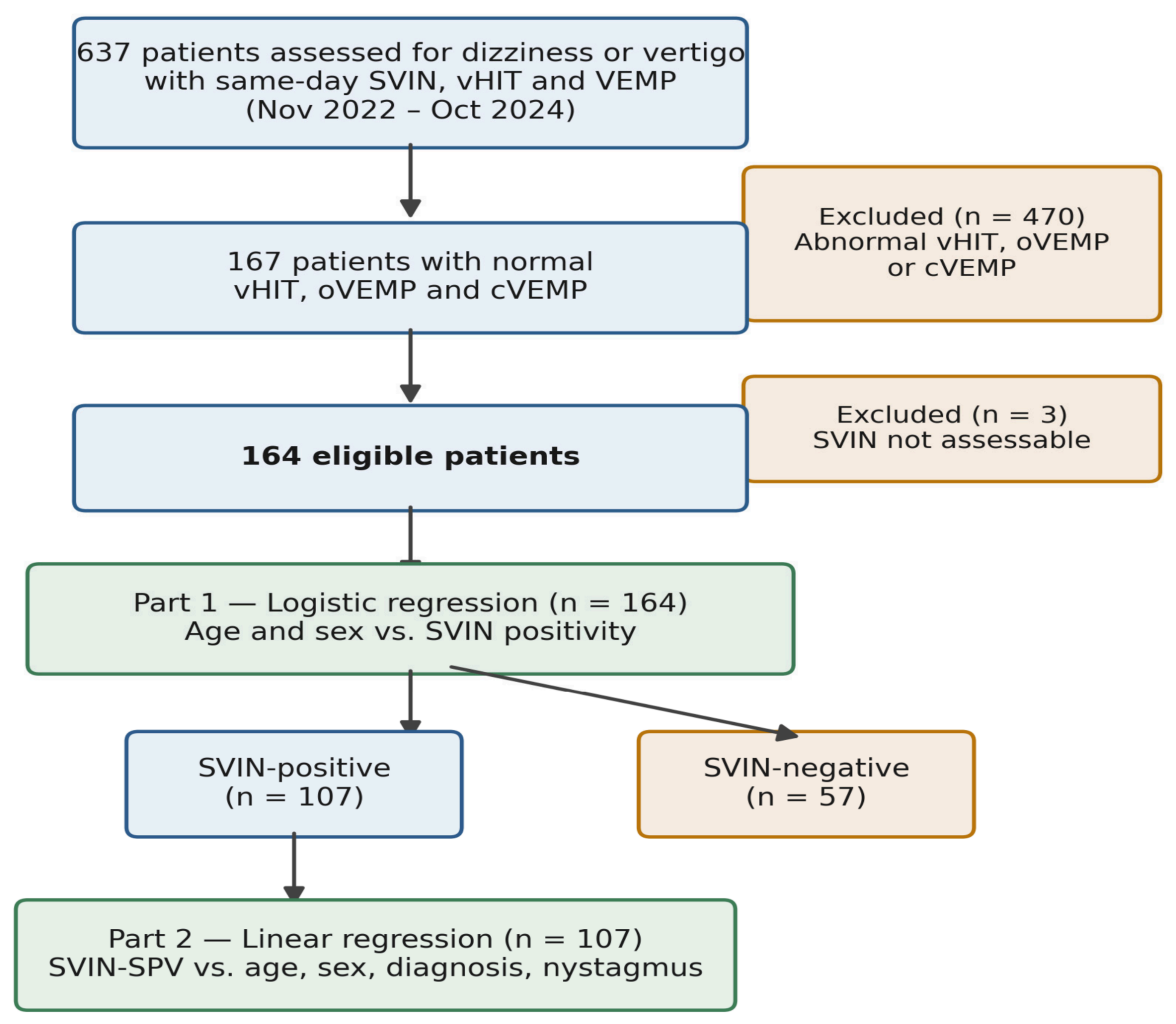
Participant flow. Of 637 patients assessed, 470 were excluded for an abnormal vHIT, oVEMP, or cVEMP (vestibular tests were required to be normal) and 3 for a non-assessable SVIN, leaving 164 eligible patients; SVIN was positive in 107 and negative in 57. Part 1 (logistic regression, n = 164) analyzed SVIN positivity; part 2 (linear regression, n = 107) analyzed SVIN-SPV intensity.

Of 637 patients assessed during the study period (1 November 2022 to 31 October 2024), 470 were excluded for an abnormal vHIT, oVEMP, or cVEMP (all three vestibular tests were required to be normal), and 3 for a non-assessable SVIN, leaving 164 eligible patients; of these, 107 had a positive SVIN and 57 a negative SVIN. All 164 eligible patients were used for the analysis of SVIN positivity, and the 107 SVIN-positive patients for the analysis of SVIN-SPV intensity (Figure 1).

The inclusion criteria were a complete otoneurological examination—including videonystagmography (VNG), vHIT, VEMPs, and SVIN performed on the same day—together with normal ocular alignment in primary gaze, no significant deviation on cover testing, and adequate visual acuity for testing. The exclusion criteria were: (1) an incomplete vestibular test battery; (2) abnormal vHIT (horizontal vestibulo-ocular reflex gain < 0.8 or vertical gain < 0.7, or the presence of reproducible refixation saccades); (3) abnormal VEMPs (interaural asymmetry ratio ≥ 40% for oVEMPs or cVEMPs, or bilaterally absent reproducible responses); (4) clinical or instrumental evidence of central vestibulopathy; (5) significant visual impairment preventing fixation; and (6) inability to sustain sufficient sternocleidomastoid contraction for cVEMP recording.

### 2.3. Vestibular Assessment

*Nystagmus evaluation.* Eye movements were recorded using video-oculography (VisualEyes 525; Interacoustics A/S, Middelfart, Denmark). Horizontal and vertical components were calibrated and analyzed. Spontaneous nystagmus (SN) was recorded in primary and lateral gaze, with and without fixation, for 30 s per condition, and its direction and slow-phase velocity (SPV) were noted. For analysis, spontaneous nystagmus was treated as a categorical variable (present/absent) and fixation suppression as effective or ineffective. Spontaneous nystagmus was considered suppressed when visual fixation reduced its slow-phase velocity by more than 75% relative to the fixation-denied condition, or abolished it completely.

*SVIN protocol.* SVIN was elicited using a 100 Hz hand-held vibrator (VVIB 100; Synapsys, Marseille, France) with the patient seated and fixation denied [6,15]. Each mastoid was stimulated once for 10 s (10 s over the right mastoid and 10 s over the left). Spontaneous nystagmus was first quantified on a separate video-oculography recording with fixation denied; its slow-phase velocity (0 deg/s when absent) was recorded. SVIN was then recorded on a separate video-oculography acquisition. For each stimulation, the slow-phase velocity was measured as its magnitude in degrees per second (unsigned), and the reported SVIN-SPV is the mean of the two mastoids, taken as such during vibration without subtraction of any baseline spontaneous nystagmus. Nystagmus was considered present when it was reproducible, stimulus-locked, and consistent in direction after both right and left stimulation; blink and goggle-slippage artifacts were excluded on offline review. Because the SVIN value was not corrected for baseline spontaneous nystagmus, the potential contribution of the latter was addressed analytically (see Statistical Analysis and Results). SVIN-SPV was analyzed as a continuous variable and secondarily stratified into three clinical bands based on previously published reference values for vibration-induced nystagmus [2,16]: normal (<2.8 deg/s), intermediate or gray zone (2.8–4 deg/s), and pathological (>4 deg/s).

*vHIT.* Horizontal and vertical semicircular canal function was tested using the vHIT (Otometrics, Taastrup, Denmark). Vestibulo-ocular reflex (VOR) gain was calculated from eye and head velocity during passive head impulses. The vHIT was considered normal when horizontal VOR gain was ≥0.8 and vertical gain ≥ 0.7, with no reproducible refixation saccades.

*VEMPs.* Ocular (oVEMP) and cervical (cVEMP) VEMPs were recorded on an Eclipse system (Interacoustics, Middelfart, Denmark), with electrode impedances kept below 5 kΩ. Stimuli were 500 Hz air-conducted tone bursts delivered monaurally through insert earphones without masking; 200 sweeps were averaged per trace at a rate of 5.1 stimuli/s over a −20 to 80 ms window; the input amplifier used a 10 Hz high-pass (6 dB/oct) and a 1000 Hz low-pass filter. For cVEMP, the active (non-inverting) electrode was placed over the upper–middle third of the sternocleidomastoid muscle, the reference over the sternum/sternoclavicular joint and the ground on the forehead; stimuli were presented at 105 dB nHL in rarefaction (rise/fall 2 cycles/4.0 ms, plateau 2 cycles/4.0 ms) with active EMG monitoring (25–70 μV) and amplitude scaling for asymmetry. The p13–n23 complex was analyzed between 10 and 25 ms, with normal latency ranges of 10–15 ms (p13) and 20–28 ms (n23). For oVEMP, the active electrode was placed on the infraorbital margin over the inferior oblique muscle (with the patient looking upward), the reference on the lateral aspect of the nose, and the ground on the forehead; stimuli were presented at 105 dB in condensation, without EMG control. The n10–p15 complex was analyzed between 5 and 25 ms, with normal latency ranges of 7–11 ms (n10) and 14–18 ms (p15). A VEMP was considered normal when reproducible waveforms were present with latencies within these ranges and an interaural asymmetry ratio < 40%.

### 2.4. Statistical Analysis

The analysis had two parts. First, to address outcome-dependent selection, a multivariable logistic regression across all 164 eligible patients tested whether age and sex were associated with the presence of a positive SVIN. Second, among the 107 SVIN-positive patients, a multivariable linear regression model was fitted with SVIN-SPV as the outcome and age, sex, ICVD status, and presence of spontaneous nystagmus as covariates.

The primary aim of the regression modeling was to assess whether age explained SVIN-SPV variability independently of diagnostic category and spontaneous nystagmus, rather than to develop a predictive model. Regression diagnostics were examined and confirmed acceptable model behavior: homoscedasticity (Breusch–Pagan *p* = 0.23), absence of autocorrelation (Durbin–Watson = 1.74), and no dominant influential observation (maximum Cook’s distance 0.24). Because regression residuals departed from normality, two sensitivity analyses were performed: a robust (M-estimator) regression and a model using log-transformed SVIN-SPV. For descriptive purposes, SVIN-SPV was categorized into three bands (<2.8°/s, 2.8–4°/s, >4°/s), and age was compared across bands using one-way ANOVA with Bonferroni-corrected post hoc comparisons. The two age groups (<60 vs. ≥60 years) were compared using the Mann–Whitney U test, and the same exact *p*-value is reported in the text and the corresponding figure. The 60-year cut-off and the velocity bands were pre-specified on the basis of prior laboratory reference thresholds. A two-tailed *p*-value < 0.05 (after correction) was considered statistically significant. All analyses were performed using Wizard 2 (version 2.0.16; Evan Miller, Chicago, IL, USA).

## 3. Results

A total of 164 patients were eligible for the analysis of SVIN positivity, of whom 107 constituted the SVIN-positive subgroup used for the intensity analysis. In this SVIN-positive subgroup (mean age 49.7 ± 14.68 years, range 14–78; 58 women, 54.2%), vestibular syndromes, classified according to the Bárány Society criteria, were spontaneous (n = 45, 42.1%), positional (n = 31, 29.0%), chronic (n = 25, 23.4%) and acute (n = 6, 5.6%). The most frequent etiological diagnoses were benign paroxysmal positional vertigo, recurrent vertigo, instability, vestibular migraine and [17] Ménière’s disease. A definite or probable diagnosis of an internationally classified vestibular disorder (ICVD) was assigned to 67 patients (62.6%). Spontaneous nystagmus was present in 56 patients (52.3%). Cohort characteristics are summarized in Table 1.

Across all 164 eligible patients (107 SVIN-positive, 57 SVIN-negative), the two groups had similar ages (49.7 ± 14.7 vs. 46.5 ± 17.1 years) but differed in sex distribution, with fewer women among SVIN-positive patients (54.2% vs. 73.7%, *p* = 0.02; Table 2). In multivariable logistic regression, age was not associated with the presence of a positive SVIN (odds ratio 1.01 per year, 95% CI 0.99–1.03, *p* = 0.28; 65% positive in patients < 60 years vs. 67% in those ≥60 years), whereas male sex was associated with a higher probability of a positive SVIN (odds ratio 2.31, 95% CI 1.14–4.68, *p* = 0.020). Age was not significantly associated with SVIN positivity in the fitted logistic model; the association between age and SVIN-SPV was therefore observed conditionally among SVIN-positive patients. We found no statistical evidence that age was associated with whether SVIN was present, although a non-significant result does not exclude a small effect (Table 3).

SVIN-SPV values were generally low, consistent with the inclusion of patients with normal vHIT and VEMP responses; the mean SVIN-SPV was 3.0°/s (range 1–11).

**Figure 2:**
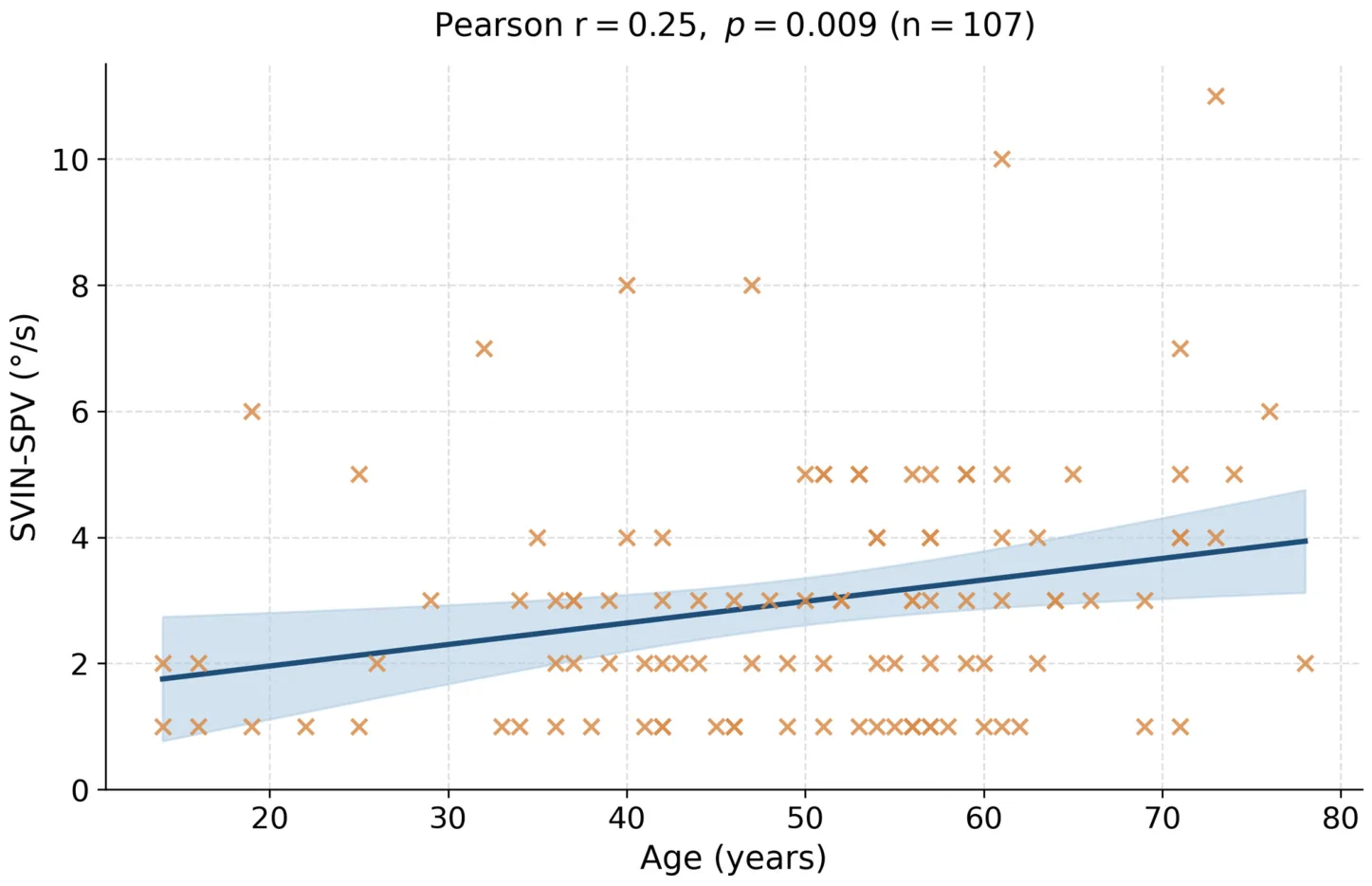
Age versus SVIN-SPV. Scatterplot of individual patient values (n = 107). The solid line represents the regression fit and the shaded area the 95% confidence interval.

SVIN-SPV correlated positively with age (Pearson r = 0.25, *p* = 0.009; Spearman ρ = 0.25, *p* = 0.009), corresponding to an unadjusted R^2^ of 0.06. In multivariable linear regression, age was the only covariate showing a statistically significant association with SVIN-SPV (β = 0.035°/s per year, 95% CI 0.009–0.061, *p* = 0.010); sex (*p* = 0.95), ICVD status (*p* = 0.99) and presence of spontaneous nystagmus (*p* = 0.70) were not associated (model R^2^ = 0.064, adjusted R^2^ = 0.027; Table 4). The age effect was confirmed by robust regression (β = 0.033, *p* = 0.002) and after log-transformation of SVIN-SPV (*p* = 0.009). These findings are presented in Figure 2.

**Figure 3:**
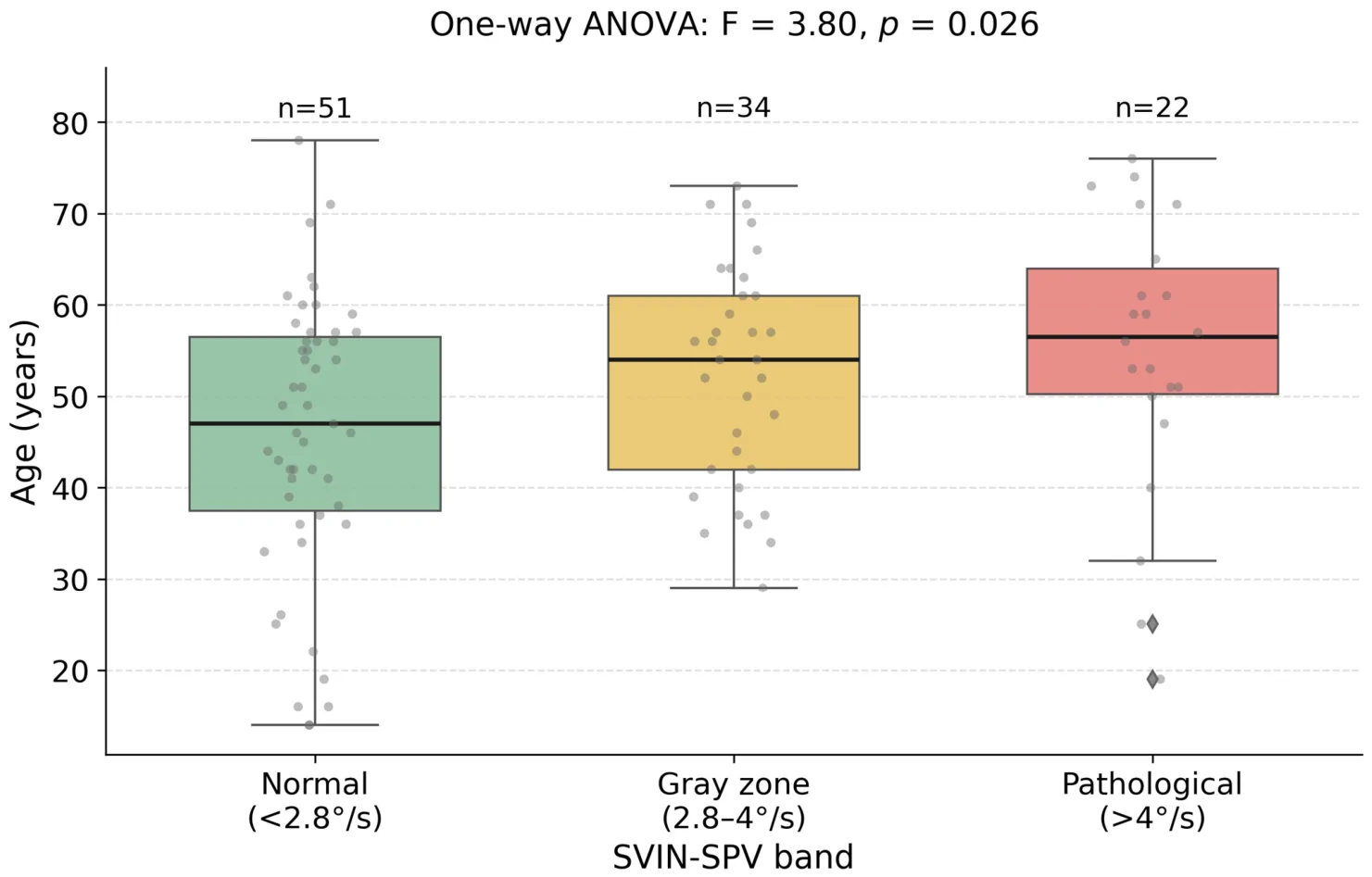
Distribution of age across SVIN-SPV bands (normal < 2.8°/s, n = 51; intermediate 2.8–4°/s, n = 34; pathological > 4°/s, n = 22). Age increased across bands (one-way ANOVA F = 3.80, *p* = 0.026).

For descriptive purposes, SVIN-SPV values were categorized into three velocity bands: normal < 2.8°/s (n = 51, 47.7%; mean age 45.8 years), intermediate 2.8–4°/s (n = 34, 31.8%; mean age 52.2 years) and pathological > 4°/s (n = 22, 20.6%; mean age 54.7 years). Mean age increased across the bands (one-way ANOVA F = 3.80, *p* = 0.026), with a significant Bonferroni-corrected difference between the normal and pathological bands. These distributions are shown in Figure 3.

**Figure 4:**
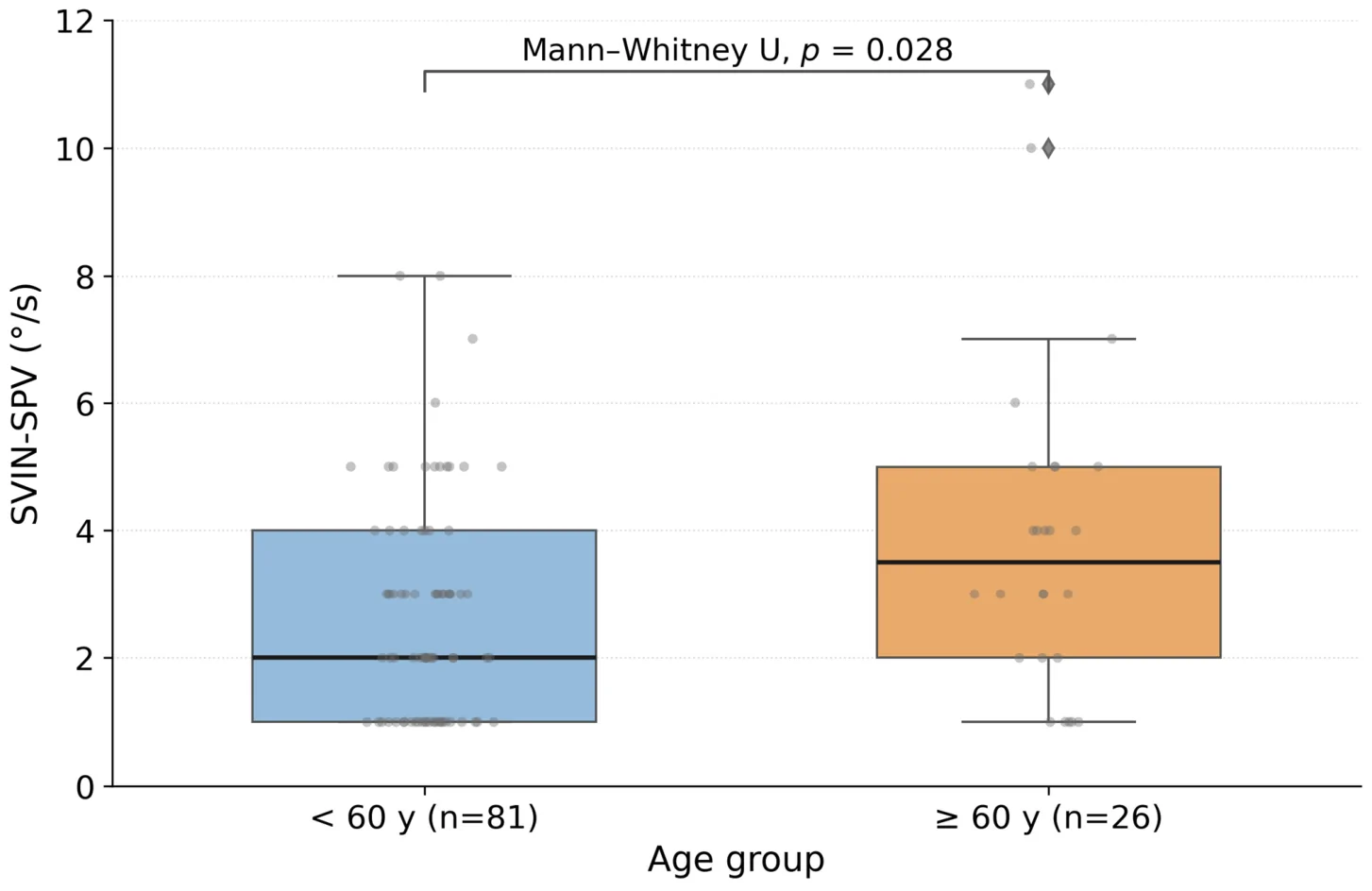
Median SVIN-SPV (interquartile range, whiskers and outliers) by age group in patients younger than 60 years (n = 81) and those aged 60 years or older (n = 26). The panel is a boxplot showing the median and interquartile range; the displayed *p*-value (Mann–Whitney U, *p* = 0.028) matches the text.

**Figure 5:**
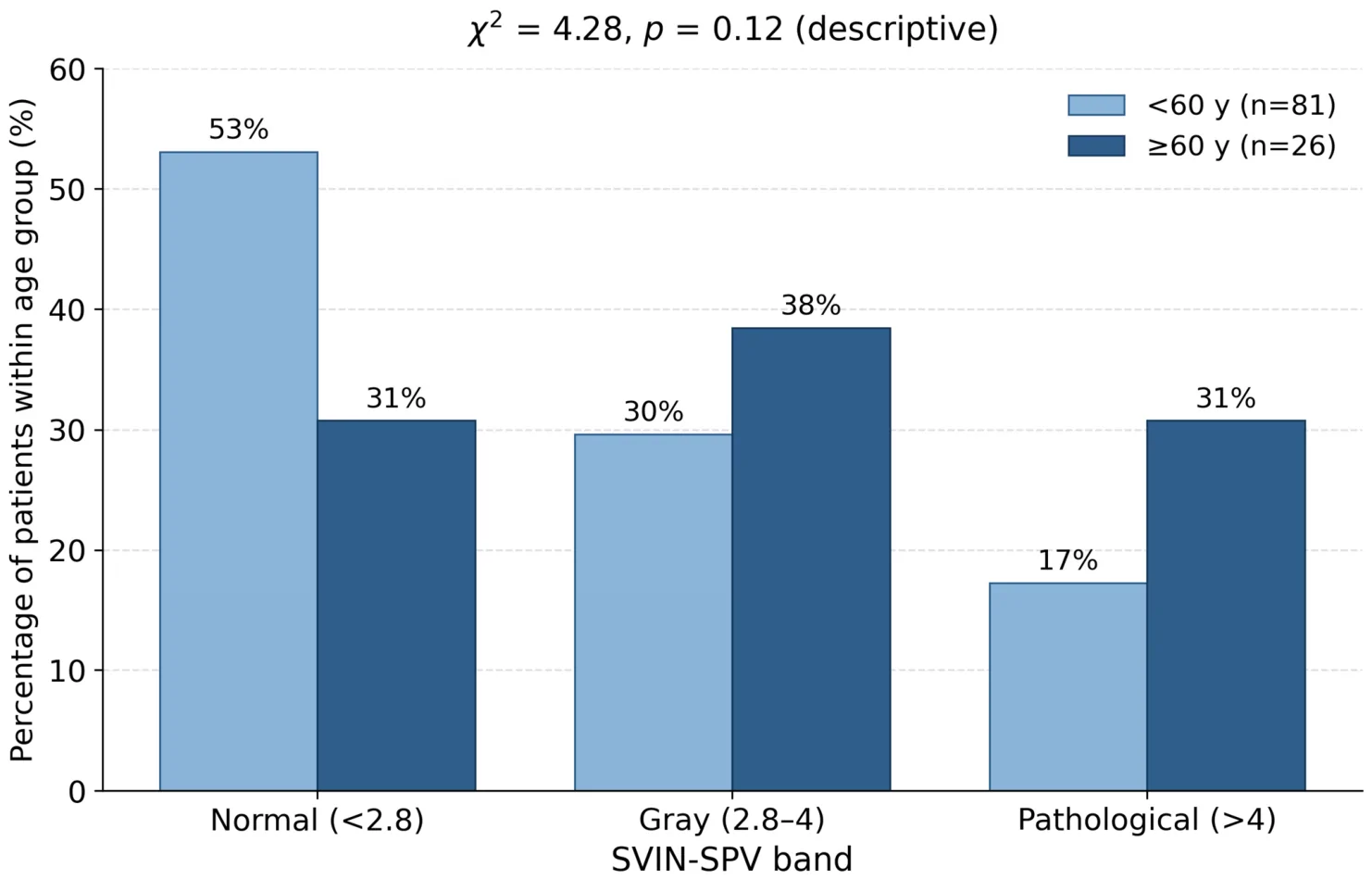
Distribution of SVIN-SPV bands (normal < 2.8°/s, intermediate 2.8–4°/s, pathological > 4°/s) by age group (<60 vs. ≥60 years), expressed as the percentage of patients within each age group. Differences were descriptive (χ^2^ *p* = 0.12).

In age-stratified analyses, patients aged ≥60 years (n = 26) exhibited higher SVIN-SPV than those aged <60 years (n = 81) (median 3.5 [IQR 2–5] vs. 2.0 [IQR 1–4]°/s; Mann–Whitney U *p* = 0.028). The distribution across SVIN-SPV bands also differed between age groups: among patients < 60 years, 53% were in the normal band and 17% in the pathological band, whereas among patients ≥ 60 years, 31% were normal and 31% pathological (χ^2^ *p* = 0.12, descriptive; Figure 4 and Figure 5).

Because SVIN-SPV was not corrected for baseline spontaneous nystagmus, we verified that the latter did not confound the age effect: baseline spontaneous-nystagmus slow-phase velocity was unrelated to age (r = 0.01, *p* = 0.94) and to SVIN-SPV (r = 0.03, *p* = 0.78), and adding it to the model left the age coefficient unchanged (β = 0.034, *p* = 0.010). In a sensitivity analysis restricted to the 51 patients without spontaneous nystagmus—in whom the SVIN-SPV outcome cannot be affected by any baseline nystagmus—the association with age remained and was somewhat stronger (Pearson r = 0.35, *p* = 0.013; β = 0.051°/s per year, 95% CI 0.011–0.091, *p* = 0.013), confirming that the age effect is not driven by baseline spontaneous nystagmus. The inclusion of the diagnostic category in a separate sensitivity model did not materially alter the association between age and SVIN-SPV. An exploratory age × diagnostic-category interaction suggested a stronger age effect among ICVD-positive patients, although this did not reach significance (*p* = 0.088).

In the BPPV subgroup (n = 31), tested in the seated position outside an active positional episode, SVIN-SPV did not differ from the rest of the cohort (median 3.0 vs. 3.0°/s; Mann–Whitney U *p* = 0.74), and the age–SVIN-SPV association was in the same direction, though not significant in this smaller subgroup (r = 0.27, *p* = 0.15; Table 5).

## 4. Discussion

In this selected clinical population with normal vHIT and VEMP responses, age was the only covariate showing a statistically significant association with SVIN-SPV in the fitted model. Sex, ICVD status, and presence of spontaneous nystagmus did not reach significance. Because a non-significant result does not establish the absence of an effect, these covariates should be regarded as not demonstrably associated in this sample rather than as having no influence. These results are consistent with prior evidence that SVIN can persist in compensated or subclinical vestibular conditions and does not depend strictly on overt unilateral vestibular loss [1,2,7]. The correlation was modest (r = 0.25; R^2^ = 0.06), so age accounted for only a small fraction of the observed variance in SVIN-SPV.

A key strength of the present analysis is its two-part design, which separates the occurrence of SVIN from its intensity. Across the full eligible cohort of 164 patients, age was not significantly associated with the presence of a positive SVIN (odds ratio 1.01 per year, *p* = 0.28), with similar positivity rates below and above 60 years. This finding is relevant for two reasons. First, because we found no statistical evidence that age was associated with SVIN positivity, the age–SVIN-SPV association observed among positive patients is less likely to be explained by outcome-dependent selection on age, although a non-significant test cannot exclude a small selection effect. Second, it suggests that aging modulates the magnitude of an existing vestibular asymmetry rather than its mere presence, which is consistent with the interpretation of SVIN as a graded marker of interlabyrinthine imbalance rather than a binary sign. The association of male sex with a higher probability of a positive SVIN (odds ratio 2.31, *p* = 0.02) was an unexpected secondary finding; whether it reflects true sex differences in vestibular asymmetry, in susceptibility to vibratory stimulation, or residual confounding cannot be determined from these data and warrants confirmation in independent samples.

The positive association between age and SVIN-SPV is counterintuitive, given the well-established age-related decline in peripheral vestibular receptor function and central adaptive capacity [13]. SVIN, however, does not index absolute vestibular drive; rather, it has been proposed to reflect interlabyrinthine asymmetry under bilateral vibratory stimulation and the efficiency of the central mechanisms that may suppress this asymmetry [9,18]. With advancing age, asymmetric degeneration of vestibular structures together with less effective central compensation may amplify measurable SVIN responses even when overall vestibular function remains within normal limits on standard tests [19,20].

This interpretation is consistent with, but not demonstrated by, experimental and clinical evidence that vestibular compensation may become slower and less complete with age. We hypothesize that vibratory stimulation could unmask residual asymmetries that remain clinically silent during physiological head movements [21]; under this hypothesis, a higher SVIN-SPV in older individuals might reflect a reduced capacity to dampen asymmetry-driven ocular-motor output rather than increased vestibular responsiveness [22]. However, our cross-sectional design cannot establish this mechanism, and unmeasured confounding cannot be excluded.

SVIN occupies a distinctive position among vestibular tests. Unlike caloric testing, which probes very-low-frequency semicircular canal function, or the vHIT, which assesses high-frequency angular-acceleration responses, SVIN relies on bilateral, non-physiological vibratory stimulation that preferentially reveals interlabyrinthine differences [1,5]. It may serve as a complementary marker of residual asymmetry and central compensation—particularly in patients with normal vHIT and VEMP responses—although vestibular-deficit severity and central compensation were not independently measured in this study [3].

The relatively low SVIN-SPV values in this cohort reflect the deliberate exclusion of patients with overt unilateral vestibular loss and the inclusion of individuals with normal vHIT and VEMP responses. Previous work has shown that SVIN responses shift with lesion severity but rarely disappear completely, supporting the view that SVIN can persist in compensated states [7,8,23]. Within this low-amplitude range, the distribution of SVIN-SPV values—and their shift toward higher bands with increasing age—appeared more informative than absolute velocity thresholds. The intermediate “gray zone” (2.8–4°/s) likely reflects overlap between physiological aging and subclinical vestibular asymmetry and should not be dismissed as clinically irrelevant, particularly in older patients [12].

Although these findings intersect conceptually with the framework of presbyvestibulopathy, SVIN-SPV should not be interpreted as a diagnostic marker for that condition [14]. SVIN may instead be viewed as a functional correlate of age-related vestibular vulnerability, capturing asymmetries that precede or accompany vestibular aging yet remain undetected by conventional high- and low-frequency vestibular tests [13,24].

This study has several limitations. The retrospective, cross-sectional design precludes causal inference, and no independent measure of vestibular-deficit severity or symptom burden was incorporated. Only SVIN-positive patients with normal vHIT and VEMP were analyzed, which introduces a selection that must be considered when interpreting the SVIN-SPV distribution. The population comprised symptomatic patients referred for vestibular testing, not healthy adults, so the findings cannot be generalized to physiological vestibular aging in the general population. The mean age was relatively low, and few patients were older than 70 years, which limits inference at the upper age range; a prospective sample enriched with older patients would strengthen the age analysis. Central vestibulopathy was excluded on clinical and instrumental grounds, but systematic neuroimaging was not performed in all patients, so a residual central contribution cannot be fully excluded. The narrow range of SVIN-SPV values limits extrapolation to acute or severe unilateral vestibular disorders. The distribution of SVIN-SPV was right-skewed; accordingly, Spearman correlation and robust and log-transformed regression models were reported and were concordant with the primary analysis. The exploratory age × diagnostic-category interaction (*p* = 0.088) was likely underpowered. Caloric testing and detailed covert-saccade analysis were not systematically available. Prospective studies integrating SVIN with caloric asymmetry and high-frequency oVEMP/vHIT metrics may further clarify its clinical role [15,25].

## 5. Conclusions

In patients with normal vHIT and VEMP, the presence of a positive SVIN was associated with male sex but not with age, whereas among SVIN-positive patients, age was associated with SVIN-SPV intensity. In this selected clinical population with normal vHIT and VEMP responses, SVIN-SPV showed a modest positive association with age, independent of diagnostic category and spontaneous nystagmus within the limits of the model. The data are compatible with possible age-related changes in vestibular asymmetry or central compensation, although the underlying mechanism was not directly assessed and cannot be established by this cross-sectional design. Given the modest effect size and the selected population, SVIN should be interpreted alongside, rather than in place of, established vestibular tests, and these findings should not yet be generalized to physiological vestibular aging in the general population.

## Figures and Tables

**Table 1:** Demographic, clinical, and vestibular characteristics of the SVIN-positive subgroup (n = 107).

Variable	n or Value	% or Range
**Demographics**		
Total patients	107	—
Age, years—mean ± SD	49.7 ± 14.68	14–78
Female sex	58	54.2%
Male sex	49	45.8%
**Age groups**		
<60 years	81	75.7%
≥60 years	26	24.3%
**Vestibular syndrome (Bárány Society classification)**		
Spontaneous vestibular syndrome	45	42.1%
Positional vestibular syndrome	31	29.0%
Chronic vestibular syndrome	25	23.4%
Acute vestibular syndrome	6	5.6%
**Internationally classified vestibular disorder (ICVD) status**		
ICVD-positive (definite or probable Bárány Society diagnosis)	67	62.6%
ICVD-negative (non-classifiable)	40	37.4%
**Spontaneous nystagmus**		
Present	56	52.3%
Absent	51	47.7%
**SVIN slow-phase velocity (SVIN-SPV)**		
Mean SVIN-SPV, °/s	3.0	1–11
SVIN-SPV band, normal (<2.8°/s)	51	47.7%
SVIN-SPV band, gray zone (2.8–4°/s)	34	31.8%
SVIN-SPV band, pathological (>4°/s)	22	20.6%

SVIN-SPV = skull vibration-induced nystagmus slow-phase velocity; SD = standard deviation; ICVD = internationally classified vestibular disorder (Bárány Society or equivalent guideline criteria).

**Table 2:** Comparison of the SVIN-positive and SVIN-negative groups within the eligible cohort (n = 164). All patients had normal vHIT, oVEMP and cVEMP.

Variable	SVIN-Positive (n = 107)	SVIN-Negative (n = 57)	*p*
Age, years (mean ± SD)	49.7 ± 14.7	46.5 ± 17.1	0.12
Age ≥ 60 years, n (%)	26 (24.3)	13 (22.8)	0.98
Female sex, n (%)	58 (54.2)	42 (73.7)	0.02

SD = standard deviation. Age compared by Mann–Whitney U test; categorical variables by chi-square test. SVIN-SPV, ICVD status and spontaneous nystagmus are not applicable to the SVIN-negative group and were not compared.

**Table 3:** Logistic regression model for the presence of a positive SVIN in the full eligible cohort (n = 164; 107 SVIN-positive, 57 SVIN-negative). Reference categories: female sex. Model fit: McFadden pseudo-R^2^ = 0.034, likelihood-ratio *p* = 0.026, AIC = 210.6.

Predictor	OR	SE (β)	95% CI	*p*
Age (per year)	1.01	0.011	0.99–1.03	0.28
Sex (male vs. female)	2.31	0.359	1.14–4.68	0.020

OR = odds ratio; SE (β) = standard error of the regression coefficient; CI = confidence interval. The positivity model was restricted to age and sex because ICVD status and spontaneous nystagmus were not available for the SVIN-negative group.

**Table 4:** Multivariable linear regression for SVIN-SPV (n = 107). Model R^2^ = 0.064, adjusted R^2^ = 0.027. Reference categories: female sex, ICVD-negative, spontaneous nystagmus absent.

Predictor	β (°/s)	SE	95% CI	*p*
Age (years)	0.035	0.013	0.009 to 0.061	0.010
Sex (male vs. female)	−0.026	0.383	−0.786 to 0.734	0.947
ICVD status (positive vs. negative)	0.005	0.397	−0.781 to 0.792	0.989
Spontaneous nystagmus (present vs. absent)	−0.147	0.385	−0.911 to 0.618	0.704

β = unstandardized regression coefficient; SE = standard error; CI = confidence interval; ICVD = internationally classified vestibular disorder. The age effect was confirmed by robust regression (β = 0.033, *p* = 0.002) and after log-transformation (*p* = 0.009).

**Table 5:** Comparison of benign paroxysmal positional vertigo (BPPV) patients and the remaining cohort. SVIN was recorded in the seated position outside an active positional episode.

Variable	BPPV (n = 31)	Others (n = 76)	*p*
Age, years (mean ± SD)	52.5 ± 12.6	48.5 ± 15.4	0.23
Female sex, n (%)	16 (51.6)	42 (55.3)	0.73
Age ≥ 60 years, n (%)	9 (29.0)	17 (22.4)	0.46
Spontaneous nystagmus, n (%)	14 (45.2)	42 (55.3)	0.34
SVIN-SPV, °/s (mean ± SD)	3.1 ± 2.2	2.9 ± 1.9	0.74
SVIN-SPV, °/s (median [IQR])	3.0 [1.5–4.0]	3.0 [1.0–4.0]	0.74
SVIN-SPV band, n (normal/gray/path)	15/9/7	36/25/15	—
Age–SVIN-SPV correlation (Pearson r)	0.27 (*p* = 0.15)	0.24 (*p* = 0.04)	—

SVIN-SPV = skull vibration-induced nystagmus slow-phase velocity; IQR = interquartile range. Continuous variables compared by Mann–Whitney U test, categorical by chi-square test.

## Data Availability

The de-identified individual-level data supporting the findings of this study are available from the corresponding author upon reasonable request, subject to institutional and ethical restrictions.

## References

[1] Dumas G., Curthoys I.S., Lion A., Perrin P.P., Schmerber S. (2017). The Skull Vibration-Induced Nystagmus Test of Vestibular Function—A Review. Front. Neurol..

[2] Perez N. (2003). Vibration Induced Nystagmus in Normal Subjects and in Patients with Dizziness. A Videonystagmography Study. Rev. Laryngol. Otol. Rhinol..

[3] García-Zamora E., Araújo P.E., Pérez-Guillén V., Vargas-Gamarra M.F., Fornés-Ferrer V., Courel-Rauch M.C., Pérez-Garrigues H. (2018). Parameters of Skull Vibration-Induced Nystagmus in Normal Subjects. Eur. Arch. Otorhinolaryngol..

[4] Marcos-Alonso S.M., Batuecas-Caletrío A. (2024). Clinical Advancements in Skull Vibration-Induced Nystagmus (SVIN) over the Last Two Years: A Literature Review. J. Clin. Med..

[5] Fabre C., Tan H., Dumas G., Giraud L., Perrin P.P., Schmerber S. (2021). Skull Vibration Induced Nystagmus Test: Correlations with Semicircular Canal and Otolith Asymmetries. Audiol. Res..

[6] Dumas G., Quatre R., Schmerber S. (2021). How to Do and Why Perform the Skull Vibration-Induced Nystagmus Test. Eur. Ann. Otorhinolaryngol. Head Neck Dis..

[7] Nuti D., Mandalà M. (2005). Sensitivity and Specificity of Mastoid Vibration Test in Detection of Effects of Vestibular Neuritis. Acta Otorhinolaryngol. Ital..

[8] Marcos-Alonso S., Ayerve N.A., Monandi Roca C., Cuesta Touma G., De Dios J.C., Sánchez-Gómez H., Santa Cruz Ruíz S., Batuecas-Caletrío Á. (2023). Use of Skull Vibration-Induced Nystagmus in the Follow-Up of Patients with Ménière Disease Treated with Intratympanic Gentamicin. Clin. Exp. Otorhinolaryngol..

[9] Hamann K.F., Schuster E.M. (1999). Vibration-Induced Nystagmus—A Sign of Unilateral Vestibular Deficit. ORL J. Otorhinolaryngol. Relat. Spec..

[10] Dumas G., Karkas A., Perrin P., Chahine K., Schmerber S. (2011). High-Frequency Skull Vibration-Induced Nystagmus Test in Partial and Total Unilateral Peripheral Vestibular Lesions. Otol. Neurotol..

[11] Zhang Y., Chen Z., Zhao H., Shen J., Zhong B., Wu Q., Yang J., Jin Y., Zhang Q., Ren P. (2022). B81 Bone Vibrator-Induced Vestibular-Evoked Myogenic Potentials: Normal Values and the Effect of Age. Front. Neurol..

[12] Neri G., Neri L., Xhepa K., Mazzatenta A. (2022). Is Skull-Vibration-Induced Nystagmus Modified with Aging?. Audiol. Res..

[13] Allen D., Ribeiro L., Arshad Q., Seemungal B.M. (2016). Age-Related Vestibular Loss: Current Understanding and Future Research Directions. Front. Neurol..

[14] Agrawal Y., Van de Berg R., Wuyts F., Walther L., Magnusson M., Oh E., Sharpe M., Strupp M. (2019). Presbyvestibulopathy: Diagnostic Criteria. Consensus Document of the Classification Committee of the Bárány Society. J. Vestib. Res..

[15] Brudasca I., Vassard-Yu G., Fieux M., Tournegros R., Dumas O., Dumas G., Tringali S. (2024). Vestibular Assessment with the vHIT and Skull Vibration-Induced Nystagmus Test in Patients with Nonprogressive Vestibular Schwannoma. J. Clin. Med..

[16] Park H.J., Shin J.E., Shim D.B. (2007). Mechanisms of Vibration-Induced Nystagmus in Normal Subjects and Patients with Vestibular Neuritis. Audiol. Neurotol..

[17] Bisdorff A., Von Brevern M., Lempert T., Newman-Toker D.E. (2009). Classification of Vestibular Symptoms: Towards an International Classification of Vestibular Disorders. J. Vestib. Res..

[18] Karlberg M., Aw S.T., Black R.A., Todd M.J., MacDougall H.G., Halmagyi G.M. (2003). Vibration-Induced Ocular Torsion and Nystagmus after Unilateral Vestibular Deafferentation. Brain.

[19] Vidal P.P., de Waele C., Vibert N., Mühlethaler M. (1998). Vestibular Compensation Revisited. Otolaryngol. Head Neck Surg..

[20] Lacour M., Bernard-Demanze L. (2015). Interaction between Vestibular Compensation Mechanisms and Vestibular Rehabilitation Therapy: 10 Recommendations for Optimal Functional Recovery. Front. Neurol..

[21] Waissbluth S., Sepúlveda V. (2021). The Skull Vibration-Induced Nystagmus Test (SVINT) for Vestibular Disorders: A Systematic Review. Otol. Neurotol..

[22] Leigh R.J., Zee D.S. (2015). The Neurology of Eye Movements.

[23] Xie S., Guo J., Wu Z., Qiang D., Huang J., Zheng Y., Yao Q., Chen S., Tian D. (2013). Vibration-Induced Nystagmus in Patients with Unilateral Peripheral Vestibular Disorders. Indian J. Otolaryngol. Head Neck Surg..

[24] Agrawal Y., Davalos-Bichara M., Zuniga M.G., Carey J.P. (2013). Head Impulse Test Abnormalities and Influence on Gait Speed and Falls in Older Individuals. Otol. Neurotol..

[25] Zhang Y., Soper J., Lohse C.M., Eggers S.D.Z., Kaufman K.R., McCaslin D.L. (2021). Agreement between the Skull Vibration-Induced Nystagmus Test and Semicircular Canal and Otolith Asymmetry. J. Am. Acad. Audiol..

